# Neighborhood greenspace and health in a large urban center

**DOI:** 10.1038/srep11610

**Published:** 2015-07-09

**Authors:** Omid Kardan, Peter Gozdyra, Bratislav Misic, Faisal Moola, Lyle J. Palmer, Tomáš Paus, Marc G. Berman

**Affiliations:** 1 Department of Psychology, The University of Chicago, Chicago, IL, USA; 2 Institute for Clinical Evaluative Sciences, Toronto, ON, Canada; 3 Indiana University, Bloomington, IN, USA; 4 The David Suzuki Foundation, Toronto, ON, Canada; 5 Translational Health Science, The University of Adelaide, Adelaide, SA, Australia; 6 Rotman Research Institute, University of Toronto, Toronto, ON, Canada; 7 Grossman Institute for Neuroscience, Quantitative Biology, and Human Behavior, University of Chicago

## Abstract

Studies have shown that natural environments can enhance health and here we build upon that work by examining the associations between comprehensive greenspace metrics and health. We focused on a large urban population center (Toronto, Canada) and related the two domains by combining high-resolution satellite imagery and individual tree data from Toronto with questionnaire-based self-reports of general health perception, cardio-metabolic conditions and mental illnesses from the Ontario Health Study. Results from multiple regressions and multivariate canonical correlation analyses suggest that people who live in neighborhoods with a higher density of trees on their streets report significantly higher health perception and significantly less cardio-metabolic conditions (controlling for socio-economic and demographic factors). We find that having 10 more trees in a city block, on average, improves health perception in ways comparable to an increase in annual personal income of $10,000 and moving to a neighborhood with $10,000 higher median income or being 7 years younger. We also find that having 11 more trees in a city block, on average, decreases cardio-metabolic conditions in ways comparable to an increase in annual personal income of $20,000 and moving to a neighborhood with $20,000 higher median income or being 1.4 years younger.

Many have the intuition that living near trees and greenspace is beneficial to our health. But how much could a tree in the street or a nearby neighborhood park improve our health? Here we set out to examine this very question by studying the relationship between health and neighborhood greenspace as measured with comprehensive metrics of tree canopy on the street vs. tree canopy in parks and private residences.

It is a known fact that urban trees improve air quality^1^,^2^, reduce cooling and heating energy use^3^, and make urban environments aesthetically more preferable^4^,^5^. Importantly, several studies have shown that exposure to greenspaces can be psychologically and physiologically restorative by promoting mental health^6^,^7^, reducing non-accidental mortality^8^, reducing physician assessed-morbidity^9^, reducing income-related health inequality’s effect on morbidity^10^, reducing blood pressure and stress levels^11^,^12^, reducing sedentary leisure time^13^, as well as promoting physical activity^14^,^15^. In addition, greenspace may enhance psychological and cardio-vascular benefits of physical activity, as compared with other settings^12^.

Moreover, experimental research has demonstrated that interacting with natural environments can have beneficial effects – after brief exposures - on memory and attention for healthy individuals^16^,^17^,^18^ and for patient populations^19^,^20^,^21^. In addition, having access to views of natural settings (e.g., from a home or a hospital bed) have been found to reduce crime and aggression^22^,^23^ and improve recovery from surgery^24^.

Although many studies have shown that natural environments enhance health or encourage healthy behaviors, to our knowledge, fewer studies have quantified the relationship between individual trees and health. In addition, studies have not separately estimated the treed area beside the streets and other urban greenspaces and related those variables to individuals’ health in various domains, including cardio-metabolic conditions, mental disorders and general health perception. Knowing the kind of greenspace that may be associated with health benefits would be critical when deciding the type of greenspace that should be incorporated into built environments to improve health.

The typical method for quantifying exposure to greenspace for individuals in large population studies is to use the percentage of area covered in greenspace in an individual’s neighborhood. The size of the areas and the accuracy (and also definition) of greenspace quantification vary across different studies. For example^10^, used data containing >10 m^2^ accuracy for greenspace and geographical units of 4 km^2^ on average in their study, Richardson *et al.* (2013) used >200 m^2^ accuracy for greenspace and geographical units that averaged 5 km^2^, and^7^ used the presence of public “natural” spaces in areas within a 5 km radius from schools to quantify exposure to nature for school-aged children.

In this study, we were interested in examining greenspace with lower granularity (i.e., higher geographical resolution) and quantifying associations that are specific to exposure to trees, as opposed to exposures to any greenspace, such as grass or shrubbery. Here, our definition of greenspace consisted of tree canopy only and not of urban grass or bushes (or other “natural” settings). This choice is based on the assumption that trees are the most consistent green components in an area and potentially the most important component for having beneficial effects^25^.

We also used a much higher geographical resolution for the following reasons. First, we wanted to distinguish between trees along the roads and streets versus those in domestic gardens and parks, and other open areas. To do so, we used individual tree data from the ‘Street Tree General Data’ and tree-canopy polygon data from the ‘Forest and Land Cover’ dataset to construct our greenspace variables. Both datasets came from the city of Toronto. Second, to ensure that the tree variables were less confounded by health insurance policies as well as demographic parameters (age, sex, education, and income), we used a single urban population (Toronto) in Canada, a country with a universal publically funded healthcare system that, compared with the United States, guarantees access to health-care services independent of income and/or employment status^26^. These health-care equalities facilitate the interpretation of the relationships between individual urban trees and health in this urban population. Although financial barriers may not impede access to health care services in Canada, differential use of physician services with respect to socio-economic status persist; Canadians with lower incomes and fewer years of schooling visit specialists at a lower rate than those with moderate or high incomes and higher levels of education despite the existence of universal health care^27^. In particular, we examined the relationship between tree canopy density beside the streets and in other areas such as parks and domestic gardens with an individual’s health. The health variables that we focused on were: 1) Overall health perception; 2) Presence of cardio-metabolic conditions such as hypertension, high blood glucose, obesity (both overweight and obese), high cholesterol, myocardiac infarction, heart disease, stroke, and diabetes; and 3) Mental health problems including major depression, anxiety, and addiction. Subjective self-rated health perception was chosen as one of the health outcomes because self-perception of health has been found to be related to morbidity and mortality rates and is a strong predictor of health status and outcomes in both clinical and community settings^28^,^29^,^30^.

Furthermore, on the tree variable side, we distinguished tree canopy of trees beside the street from those planted in other areas, such as parks and private backyards. A distinction of these different sources of tree canopy may be helpful for urban planning policies. We hypothesized that street trees could have stronger beneficial associations with individual’s health because they may be more accessible to all residents in a given neighborhood as residents are likely exposed to street trees in their daily activities and through views from their windows; for example see^24^.

Figure 1 shows a geographic map of the individual tree data (i.e., the individual trees on the street) and Fig. 2 shows a geographic map of the satellite tree data (i.e., the amount of tree canopy) for different neighborhoods in the city of Toronto. Both tree datasets were used to quantify the “greenness” of the neighborhoods (see Methods). Figure 3 shows the dissemination areas (i.e., Toronto neighborhood units) that were used in our analysis. The highlighted neighborhoods are the ones that were included in our analysis.

To uncover the relationships between neighborhood greenspace and health we performed two analyses. The first was a multiple regression of each health outcome on socio-economic, demographic and tree density variables. The second was a canonical correlation analysis where we examined the multivariate relationship between *all* health outcomes and socio-economic, demographic and tree density variables. Our canonical correlation model is shown in Fig. 4. In all of these analyses we attempted to quantify the independent relationships of street tree canopy and non-street tree canopy on health.

## Results

### Regression Results

#### Health Perception

Our results suggest that people who live in areas that have more (and/or larger) trees on the streets report better health perception, after controlling for demographic factors, such as income, age and education [p < 0.0001]. As can be seen in Table 1, the regression coefficient for the street tree density variable shows that a four percent square meters (400 cm^2^) increase in the treed area for every square meter of neighborhood predicts about 0.04 increased health perception (i.e., 1% of our 1–5 health perception scale) for individuals living in that area. A 400 cm^2^/m^2^ increase in treed area is equal to the addition of about 200 average trees (with 40 m^2^ crown area) on the streets in a dissemination area of almost average size (about 200,000 m^2^) in Toronto. This is approximately 10 more trees per city block (a DA usually contains about 25 blocks). As can be seen in Table 1, this increase in health perception is equivalent to the effect of a $10,200 increase in annual household income and living in a DA with equally (i.e., $10,200) higher median income. (Notice that for this comparison we added up the estimates of income and area income because a hypothetical increase of income for the families in a DA also increases the median area income in that DA to the same extent). This same increase in health perception is also, on average, equivalent to being 7 years younger.

Other than street tree density, variables that independently predict better health perception in this multiple regression are: eating more servings of vegetables and fruits in one’s diet (1 more serving per day predicts 1.2% better health perception [p < 0.0001]), being younger (10 years less age predicts 1.5% better health perception [p < 0.0001]), being male (males have on average almost 1% better health perception than females [p = 0.0004]), having higher education (belonging to one higher educational group predicts 1.6% better health perception [p < 0.0001]), living in more affluent neighborhoods (belonging to one higher area median income group predicts 0.7% better health perception [p < 0.0001]), and having higher household income (belonging to one higher income group predicts 1.6% better health perception [p < 0.0001]). It should be mentioned that the associations between health perception and tree density and other predictors reported here explain 9% of the variance in health perception. While the model explains a significant proportion of the variance in the data, it does not explain all of the variance of the dependent variable. This is true of all models whose R^2^ values are less than 1. As such the model’s predictions may not always hold true if the other unidentified factors that predict the remaining variability in health perception are not controlled for.

#### Cardio-metabolic Conditions

Results of regressing the Cardio-metabolic conditions index on the independent variables are shown in Table 2. Results suggest that people who live in areas that have more (and/or larger) trees on the streets report significantly fewer cardio-metabolic conditions. People reported decrease of 0.04 units of cardio-metabolic conditions (0.5% of the 0–8 scale for cardio-metabolic conditions) for every increase of 408 cm^2^/m^2^ in tree density. This is approximately equivalent to 11 more average-sized trees on the streets per city block. This effect for cardio-metabolic conditions is equivalent to a $20,200 increase in both area median income and annual household income adjusted for other variables. This decrease in cardio-metabolic conditions is also, on average, equivalent to being 1.4 years younger.

Other than street tree density, variables that predict fewer cardio-metabolic conditions, after controlling for other variables in this multiple regression, are: eating more servings of vegetables and fruits in one’s diet (1 more serving per day predicts 0.08% less cardio-metabolic conditions [p = 0.0129]), being younger (10 years less age predicts 3.7% less cardio-metabolic conditions [p < 0.0001]), being female (females report on average 3.3% less cardio-metabolic conditions than males [p < 0.0001]), having higher education (belonging to one higher educational group predicts 0.71% less cardio-metabolic conditions [p < 0.0001]), living in more affluent neighborhoods (belonging to one higher area median income group predicts 0.36% higher reported health perception [p < 0.0001]), and having higher household income (belonging to one higher income group predicts 0.28% less cardio-metabolic conditions [p < 0.0001]). In addition, we added the interaction terms of all predictors with the tree density variables and the models R^2^ for health perception and cardio-metabolic conditions did not improve much (ΔR^2^ = 0.0008 for health perception, ΔR^2^ = 0.0009 for cardio-metabolic conditions), even though there was a small positive interaction between street tree density and age that was statistically significant. We chose not to include these interactions due to lack of a priori hypotheses, their small effect sizes and to preserve the models simplicity. Again, it should be mentioned that the associations between cardio-metabolic conditions and tree density and other predictors reported here explain 19% of the variance in cardio-metabolic conditions. While the model explains a significant proportion of the variance in the data, it does not explain all of the variance of the dependent variable. This is true of all models whose R^2^ values are less than 1. As such the model’s predictions may not always hold true if the other unidentified factors that predict the remaining variability in cardio-metabolic conditions are not controlled for.

#### Mental Disorders and Other Disorders

Results of Mental Disorders and Other Disorders can be found in Supplemental Tables S1 and S2. Regressing the Mental Disorders index on the independent variables do not capture a significant amount of variance in Mental Disorders in the data [R^2^ = 0.0136, adjusted R^2^ = −0.0111, p = 0.1820]. We will further investigate this issue later in the canonical correlation analysis.

Finally, the Other Disorders index is not a coherent variable and was not constructed to be used as a dependent variable in the regression analyses, but mainly was constructed as a control variable for the canonical correlation analysis. Nonetheless, results of regressing the Other Disorders index (Cancer, Migraines, Arthritis, or Asthma) on the independent variables are shown in Table S2.

### Canonical Correlation Results

Figures 5, 6, 7 show the results from the canonical correlation analysis, which finds the relationship (i.e., linear combination of weights) between two sets of variables. The height of each bar shows the correlation of each variable with the corresponding set of canonical weights. Error bars show ±2 standard errors containing both between and within imputation variance calculated by bootstrapping imputed data sets. Importantly, all canonical variates are orthogonal to one another.

The canonical correlation coefficient (r) for each pair of linear composites is shown near the bidirectional arrow representing the relationship between the two sets of variables (demographic and green-space variables and health-related variables). The canonical correlation coefficients for all the four pairs of linear composites were statistically significant (p < 0.0001 for Bartlett’s approximate chi-squared statistic with Lawley’s modification).

The first pair of linear composites (Fig. 5) is dominated by the effect of age on physical disorders (Cardio-metabolic and Other disorders). This suggests that being older is highly correlated (r = 0.4565, R^2^ = 0.2084) with having more cardio-metabolic conditions, as well as cancer, arthritis, asthma and migraines.

The second pair of linear composites is mainly dominated by Health Perception and shows that individuals with higher annual income, higher education, higher vegetables/fruits consumption and who live in areas with higher street tree density report the best health perception. This replicates and extends the results found in the regression. The same group of people also reports fewer cardio-metabolic conditions, although the errorbar for the loading of these conditions crosses zero (indicating a non-significant effect). This is possibly due to the fact that the main part of the variability in cardio-metabolic conditions (that was mainly due to older age) was already captured by the first canonical loadings. The canonical correlation for this second linear composite is of medium size (r = 0.2868, R^2^ = 0.0822).

The third pair of linear composites has a modest effect size (r = 0.2216, R^2^ = 0.0491) and is mainly dominated by sex. This composite shows that females report more other disorders and more mental disorders. This complies with the regression results and the fact that occurrence of breast cancer is more frequent among women even at younger ages^31^.

Results from the fourth composite are shown in Supplementary Figure S1. The fourth component was dominated by mental disorders after much of the variability due to sex was extracted by the previous composites (mainly third composite). Neither the demographic nor the tree density variables significantly correlated with the fourth canonical scores. The very small effect size (r = 0.0539, R^2^ = 0.0029) shows that the data and variables might not be rich enough for an analysis of mental disorders, as mentioned before in the regression analysis. Indeed, only a non-reliable combination of demographic and tree variables seem to be related to more mental disorders at this stage of analysis. Future studies with more detailed data regarding mental disorders may help to test the results found for the fourth composite.

Finally, Table 3 shows the communalities for all the variables, which are computed as sum of the squared loadings across all latent variables and represent how much of the variance in the variable has been accounted for by the canonical correlation model. The communality results show that the canonical variates are able to capture/reproduce at least 15% of the variance in all original variables. In conclusion, both the regression and the canonical correlation analyses suggest that higher tree density on the streets, in a given dissemination area, correlates with better health perception and fewer cardio-metabolic conditions for people living in that dissemination area.

## Discussion

Results from our study suggest that people who live in areas with higher street tree density report better health perception and fewer cardio-metabolic conditions compared with their peers living in areas with lower street tree density. There are two important points about our results that add to the previous literature. First, the effect size of the impact of street tree density seems to be comparable to that of a number of socioeconomic or demographic variables known to correlate with better health (beyond age). Specifically, if we consider two families, one earning $10,200 more annually than the other, and living in a neighborhood with the same higher median income, it is predicted that the more affluent family who is living in the richer neighborhood perceives themselves as healthier people. Interestingly, however, that prediction could turn out to be wrong if the less affluent family lives in a neighborhood that has on average 10 more trees beside the streets in every block. Regarding cardio-metabolic conditions, the same scenario is expected to hold true for an income difference of $20,200.

Ten more trees in every block is about 4% increase in street tree density in a dissemination area in Toronto, which seems to be logistically feasible; Toronto’s dissemination areas have a 0.2% to 20.5% range of street tree density and trees can be incorporated into various planting areas along local roads. According to our findings improving health perception and decreasing cardio-metabolic conditions by planting 10 more trees per city block is equivalent to increasing the income of every household in that city block by more than $10,000, which is more costly than planting the additional 10 trees. (See the “Urban Watershed Forestry Manual, Part 3 Urban Tree Planting Guide” for estimation of urban tree planting and maintenance costs and other considerations for urban tree planting. Generally, planting and maintenance of 10 urban trees could annually cost between $300 to $5000). Finally, it should be mentioned that this estimation of increased tree density being equivalent to specific increases in economic status of people is based on respondents from Canada, which has a publically funded universal health-care system. It may be the case that in other countries that do not have universal health care individuals’ health may be more affected by economic status, which could cause the tree density relationship with health to be smaller-in economic terms. This, however, is an empirical question that is certainly worthy of further investigation.

The second important finding is that the “health” associations with tree density were not found (in a statistically reliable manner) for tree density in areas other than beside the streets and along local roads. It seems that trees that affect people most generally are those that they may have the most contact (visual or presence) with, which we are hypothesizing to be those planted along the streets. Another possible explanation could be that trees on the street may be more important to reductions in air pollution generated by traffic through dry deposition^32^. This *does not* indicate, however, that parks are not beneficial. This study only shows that planting trees along the roads may be more beneficial than planting trees in parks and private residences at least for these health measures. For example, our sample only consists of adults and trees in parks may be more beneficial to children who spend more time in such locations^33^. Future studies need to address this possibility more thoroughly.

An important issue that is not addressed in this study is the mechanisms by which these beneficial effects of proximity to more (or larger) urban trees on health occur. Improving air quality, relieving stress, or promoting physical activity could all be contributing factors to improved reported health. The current study provides two pieces of information that could be useful when trying to study the underlying mechanisms of the health benefits attained from urban trees. First, more than proximity (tree density in the neighborhood), it is the availability of the trees to the largest proportion of people (trees on the roads) that is beneficial. Second, the form of the relationship is linear, at least in the density range of 0 to 20% for trees on the streets found in the city of Toronto (i.e., adding the quadratic or the square root of street tree density to the multiple regressions did not improve the models, suggesting that the relationship of health outcomes with street tree density neither decreases (quadratic transformation), nor increases (square root transformation) in a meaningful way at higher levels of street tree density). These two results imply that: 1) some of the effects may be partially related to the mere visual exposure to trees^16^,^18^,^24^ or to the dry deposition of air pollutants and 2) that the effects are not likely to plateau or accelerate, in a meaningful way, as the level of tree canopy density increases.

In addition, in a post-hoc analysis, we compared the health outcomes of individuals living in areas with more leaf-retaining versus more deciduous trees, adjusted for street and other tree density and demographic variables. Our analysis showed that people living in year-round green areas (more leaf-retaining trees) reported less cardio-metabolic conditions (p = 0.017) than their peers, but not better health perception. Again, while not conclusive, this result points to some importance regarding the types of trees that should be planted, but it would be much too premature to favor the planting of non-deciduous vs. deciduous trees.

Our study could benefit from improvements in at least three aspects. First, we used cross-sectional data for practical reasons; longitudinal data would provide us with much stronger inferences of causality. Second, our health data items are self-reported, which introduces some error and potential biases in health variables reported. Third, we are assuming that controlling for area median income accounts for many other neighborhood variables that could affect mental and physical health in indirect ways (such as neighborhood safety, pollution, etc.), which might not always hold true. In future research we plan to test our current findings in a more comprehensive manner that obviates the mentioned limitations. In summary, our results show that street trees are associated with a significant, independent and reliable increase in health benefits in urban populations and that small increases in the number of trees along the street could improve health markedly and in cost-effective ways.

## Materials and Methods

Canada is divided into geographical units called dissemination areas (DA), which consist of 400 to 700 inhabitants and whose boundary lines mostly follow roads. We used data from 3,202 DAs located in the city of Toronto with an average population of 690 individuals and average physical size of 172,290 m^2^.

**Figure 1:**
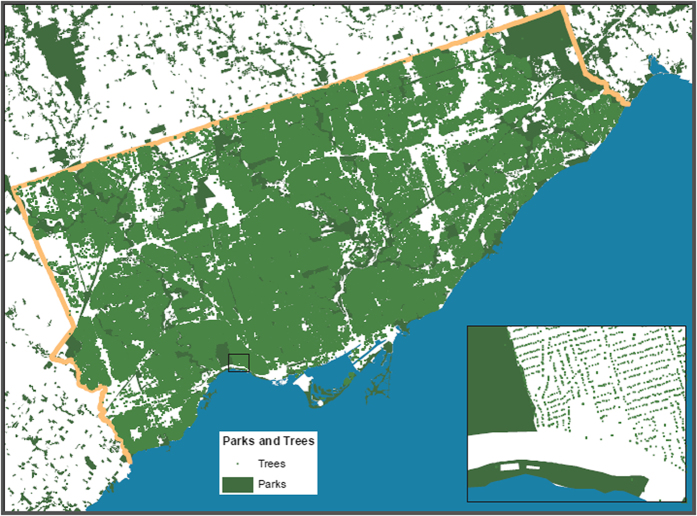
The Greenspace map of the city of Toronto constructed from the individual tree information Street Tree General Data. This image is shown in much lower resolution compared to the real image and the dissociation between individual trees and other areas is clearly perceivable for the zoomed-in area. Parks are shown in dark green. This figure was created using Environmental Systems Research Institute’s (ESRI) ArcGIS software v. 10.2.

We combined data from three different sources to construct our tree, health and demographic variables:

The first source of tree canopy data came from the ‘Street Tree General Data,’ which is a Geographical Information System (GIS) dataset that lists the locations of over 530,000 individual trees planted on public land within the city of Toronto. This dataset comes from experts who traversed the city of Toronto and recorded tree species and diameters at breast height. Trees in public parks are not listed as the listed trees were only from public land that lines the streets. The set contains each tree’s common and botanical names, their diameters at breast height (DBH), the street addresses and the general location reference information. Figure 1 shows the green-space map of Toronto generated from these data for illustration.

**Figure 2:**
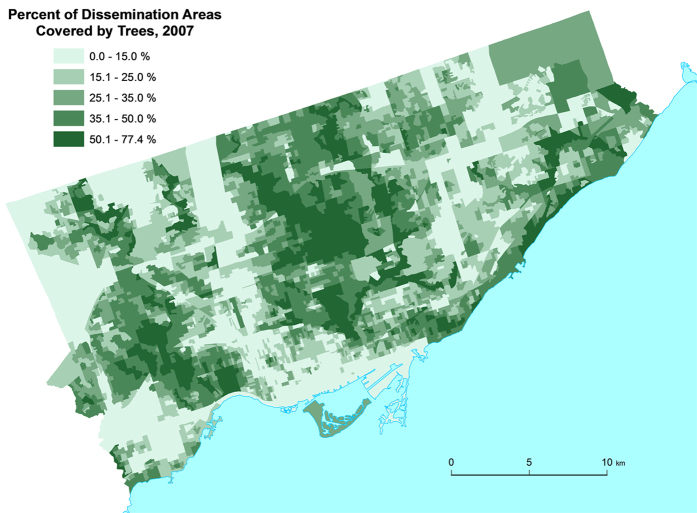
The Greenspace map of the city of Toronto constructed from the Geographical Information System (GIS) polygon data set Forest and Land Cover. The levels are shown in units of 10–15% for display purposes only as we analyzed these data as a continuous variable. This figure was created using Environmental Systems Research Institute’s (ESRI) ArcGIS software v. 10.2.

The second source of tree canopy data came from the Geographical Information System (GIS) polygon data set ‘Forest and Land Cover,’ which contained detailed areal information of tree canopies in Toronto. In these data, the satellite imagery resolution was 0.6 m – from QuickBird Satellite imagery, 2007. The treed area was calculated using automated remote sensing software - Ecognition. This automated land-cover map was then monitored by staff from the University of Vermont Spatial Analysis Lab and adjusted to increase accuracy. In this dataset there is the ability to differentiate shrub cover from trees. There is, however, some susceptibility to errors when differentiating large shrubs from trees. To validate the accuracy of the QuickBird satellite imagery, it was compared with two other methods used to assess tree canopy cover: 1) Ocular estimates of canopy cover by field crews during data collection in 2008; 2) 10,000 random point samples of leaf-off and leaf-on aerial orthophotos (imagery available in required orthorecitifed format included 1999, 2005 and 2009)^34^. The tree canopy coverage estimates for each of the respective approaches were: QuickBird: 28%; Ocular: 24%; and Aerial Orthophotos: 26.2% respectively^34^. Because of the similarity in results, we can be confident in the accuracy of the QuickBird satellite results. For more information on the automated classification of leaf-on tree canopy from the 2007 satellite imagery see Appendix 4 of^34^. Figure 2 shows a map of tree canopy in each dissemination area as generated from the QuickBird Satellite.

**Figure 3:**
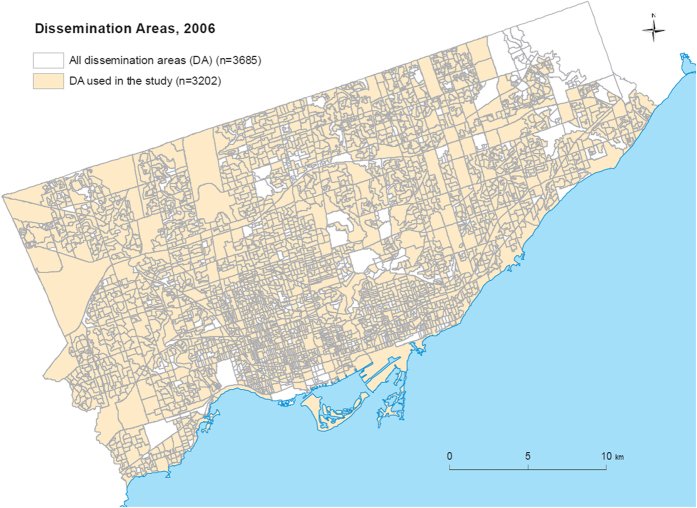
The dissemination area map of the city of Toronto (2006). The colored regions show the dissemination areas that were included in the study. This figure was created using Environmental Systems Research Institute’s (ESRI) ArcGIS software v. 10.2.

Information about individuals’ health and demographics was obtained in the context of the Ontario Health Study (https://www.ontariohealthstudy.ca). This is an ongoing research study of adults (18 years and older) living in the Canadian province of Ontario aimed at investigating risk factors associated with diseases such as: cancer, diabetes, heart disease, asthma, and Alzheimer’s Disease. The data were collected using two (similar) versions of a web-based questionnaire consisting of demographic and health-related questions. These questionnaires were completed by 94,427 residents living in the greater Toronto area between September, 2010 and January, 2013. For this study, we used data from a subset of 31,109 residents (31,945 respondents, out of which 827 were removed during quality control for having duplicate records and 9 were removed because of missing consent records). A record was considered a duplicate with the following data quality checks: 1) Multiple registrations of the same Last Name, First Name and Date of Birth 2) Multiple registrations of the same Last Name, First Name and Postal Code 3) Multiple registrations of the same Last Name, First Name, Date of Birth and Postal Code 4) Multiple registrations of the same email address. Additional data quality checks included several built-in checks in the online system, which included automatic skip patterns and limited ranges for free text numerical responses such that participant responses must be within reasonable limits. The final sample included individuals who resided in the 3,202 dissemination areas of the city of Toronto as individual tree data were only available for these areas. These dissemination areas are shown in Fig. 3.

### Demographic Variables

For each individual, we used sex (59% female; compared to the population male/female ratio: Toronto’s population was 48.0% male and 52.0% female in 2011 according to Statistics Canada), age (Mean = 43.8, range = 18–99; as of 2011 the mean age of residents above 19 years of age for the entire population of Toronto is: 47.9 according to Statistics Canada), education (coded as: 1 = none (0.0%), 2 = elementary (1.0%), 3 = high school (15.3%), 4 = trade (3.3%), 5 = diploma (15.9%), 6 = certificate (5.9%), 7 = bachelor’s (35.3%), 8 = graduate degree (23.3%), with Mean = 6.07, range = 1–8; According to the 2011 National Household Survey in www.toronto.ca, the distribution of education for the entire city of Toronto is the following: 33% of all City residents 15 years and over have a bachelor degree or higher, 69% of City residents between the ages of 25 and 64 years have a postsecondary degree, 17% of 25–64 years old residents have graduate degrees), and annual household income (coded as: 1 = less than $10 000, 2 = $10 000 – $24 999, 3 = $25 000 – $49 999, 4 = $50 000 – $74 999, 5 = $75 000 – $99 999, 6 = $100 000 – $149 999, 7 = $150 000 – $199 999, 8 = $200 000 or more, with Mean = 4.67 which is equivalent to $90 806 annual income range = 1–8; compared to the entire city of Toronto’s population mean household income, which was: $87,038 in 2010 according to Statistics Canada), as well as diet (number of fruits and vegetable servings respondent consume every day, with Mean = 2.24, range = 0–10), as potential confounding variables. In addition, for each dissemination area we used the area median income from Statistics Canada and coded those data the same as the household income data, with mean = 4.08, range = 2–8. Population densities in a given DA were used in the multiple imputation analysis but not as a variable in the regressions or the canonical correlation analyses. The correlations between demographic variables can be found in Figure S2 of Supplementary Information.

Our studied sample had similar demographics to the entire city of Toronto, but was slightly younger (mean age = 43.8; Toronto population = 47.9), slightly more female (59%; Toronto population = 52%), slightly more educated (35.3% had bachelor’s degrees vs. 33% in the Toronto population) and slightly wealthier (mean household income = $93,399 vs. $87,038 in the entire city of Toronto).

### Green-space variables

***Crown area of the trees*** was used to calculate the density of area covered by trees separately for the trees on the streets and the trees from greenspace in private locations and parks in each DA. We estimated the crown area of the trees based on their diameter at breast height (DBH) values. We obtained formulas for estimating tree crown diameter based on DBH for 8 tree types (Maple, Locust, Spruce, Ash, Linden, Oak, Cherry, and Birch) that were derived from forestry research. Forestry researchers have fit linear and non-linear models to relate crown diameter and DBH for different species of trees. These models achieved good fits as verified by their high R^2^ values (above 0.9)^35^,^36^. The formulas that were used to estimate crown diameters from DBH for these tree types and their references can be found in the Supplementary Equations section of the Supplementary Information. These 8 tree species covered 396,121 (83%) of the trees in our dataset. For the other 81,017 (17%) of the trees, we estimated crown diameter based on the linear regression of crown diameters on DBHs obtained from the 83% of the trees belonging to the tree types with known crown formulas. The crown areas of all the trees were then calculated using the crown diameters and assuming that the crown areas were circular in shape.

***Street tree density*** for each dissemination area was quantified as the total area of the crowns of trees (m^2^) beside the streets in the dissemination area over total dissemination area size (m^2^) multiplied by 100 to be in percentage format. The range for this variable was found to be from 0.02% in the areas with the least street tree density to 20.5% in the areas with highest street tree density (Mean = 4.57%). ***Other tree density*** for each dissemination area was calculated by subtracting out the area covered by crowns of the trees on the streets (street tree area) from the total treed area (m^2^) in the dissemination area (from the satellite Tree Canopy data), and then dividing that by the area size and multiplying by 100 to be in percentage format. The range for this variable was found to be from 0.00% in the areas with almost no trees in parks (or no parks), no domestic gardens or other open areas; to 75.4% in areas with high tree density and parks (Mean = 23.5%). As mentioned above, there was limited ability to differentiate large shrub cover from tree cover in the satellite data. Therefore, the variable “other tree density” could contain some unwanted large shrub cover as well, especially for areas with very high other tree density.

### Health variables

All of the health variables were constructed from the self-reported items in the Ontario Health Study (OHS). Items related to disorders were based on the question “Have you ever been diagnosed with …?” and coded with 0 = No and 1 = Yes. These consisted of physical conditions including high blood pressure, high cholesterol, high blood glucose, heart attack (MI), stroke, heart disease, migraines, chronic obstructive pulmonary disorder (COPD), liver cirrhosis, ulcerative colitis, irritable bowel disease (IBD), arthritis, asthma, cancer, and diabetes (DM), as well as mental health conditions including addiction, depression, and anxiety. About 66.3% of all respondents reported having at least one of the mentioned health conditions. The percentages of “Yes” responses for each of these conditions are reported in Supplementary Table S3. Additionally, body mass index (BMI) for each person was calculated from his/her self-reported height and weight. Our “Obesity” variable was constructed as 0 for BMI below 25, 0.5 for BMI between 25 and 30 (overweight, 26% of respondents), and 1 for BMI over 30 (obese, 13% of respondents). Other variables drawn from these data are general health perception (self-rated health (1 = poor, 2 = fair, 3 = good, 4 = very good, 5 = excellent, with Mean = 3.66, range = 1–5), and four more variables that were used in the multiple imputations to increase the accuracy of imputations: walking (the number of days a participant has gone for a walk of at least 10 minutes in length last week, with Mean = 5.33, range = 0–7), smoking (if participant has ever smoked 4-5 packs of cigarettes in their lifetime, 38% Yes), alcohol consumption frequency (coded as 0 = never, 1 = less than monthly, 2 = about once a month, 3 = two to three times a month, 4 = once a week, 5 = two to three times a week, 6 = four to five times a week, with Mean = 3.60, range = 0–7), and alcohol binge frequency (coded as 0 = never, 1 = 1 to 5 times a year, 2 = 6 to 11 times a year, 3 = about once a month, 4 = 2 to 3 times a month, 5 = once a week, 6 = 2 to 3 times a week, 7 = 4 to 5 times a week, 8 = 6 to 7 times a week, with Mean = 1.62, range = 0–8).

The dependent variables related to physical and mental health were created from the multiple-imputed data. For each complete dataset, the ***Cardio-metabolic Conditions*** index was constructed by summing the following seven variables related to cardio-metabolic health: High Blood Glucose, Diabetes, Hypertension, High Cholesterol, Myocardial infarction (heart attack), Heart disease, Stroke, and “Obesity” with Mean = 0.89, range = 0–8. The ***Mental disorders*** index was constructed by summing Major Depression, Anxiety, and Addiction, with Mean = 0.26, range = 0–3. The ***Health Perception*** index was the third dependent variable in our analyses with Mean = 3.66, range = 1–5. The ***Other disorders*** index consisted of Cancer, Migraines, Asthma, and Arthritis (Mean = 0.48, range = 0–4. This index was constructed to be a control variable in the canonical correlation analysis. The additional variables (e.g., cirrhosis) were included to increase the accuracy of the imputation, but were not analyzed. The correlation matrix between the health variables, the tree variables, and the demographic variables is reported in supplementary Figure S2 of the Supplementary Information.

### Multiple imputations analysis

The self-reported health data contained some missing values for different variables (mainly due to “I don’t know” responses). List wise deletion of the data (keeping only participants with no missing values in any of the items) would have resulted in a loss of 73% of the participants because the missing values in the different items were distributed across subjects, and was therefore an unreasonable method of analysis. To handle the missing data problem, we assumed that the data were missing at random (MAR), meaning that the probability of missingness for a variable was not dependent on the variable’s value after controlling for other observed variables. We then replaced the missing values with multiple imputed data^37^,^38^,^39^. Thirty complete datasets were created from the original dataset using the estimate and maximize (EM) algorithm on bootstrapped data implemented by the Amelia package for R [Amelia^40^;]. All of the 30 imputations converged in less than 11 iterations. Variables used in the imputations and their missing percentages are reported in Supplementary Table S4.

### Regression analysis

The regression analyses were performed separately for each imputed dataset and then combined based on Rubin’s rules^38^ using the *Zelig* program in R^41^. Rubin suggested that the mean of each regression coefficient across all imputed datasets be used as the regression coefficients for the analysis. In addition, to avoid underestimation of standard errors and taking the uncertainty of the imputed values into account, both the within imputation variance and between imputation variance of each coefficient should be used to construct the standard error for each regression coefficient. Lastly^42^, proposed using degrees of freedom estimated as a function of the within and between imputation variance and the number of multiple imputations when approximating the t-statistics for each parameter.

To assess the amount of the variance in the dependent variables that is explained by the regression model for the multiple imputed data we used the method suggested by Harel (2009) to estimate the R^2^ and the adjusted R^2^ values. Based on this method, instead of averaging R^2^ values from the 30 imputations, first the square root of the R^2^ value (r) in each of the imputed datasets is transformed to a z-score using Fisher’s r to z transformation, z = atanh(r). The average z across the imputations can then be calculated. Finally, the mean of the z values is transformed back into an R^2^. The same procedure can be used for adjusted R^2^ values. Harel (2009) suggests that the number of imputations and the sample size be large when using this method, which holds true in the current study. Also, the resulting estimates of R^2^ could be inflated (i.e. are too large), while estimates of adjusted R^2^ tend to be biased downwards (i.e. are too small). Therefore, we estimated both values for a better evaluation of the explained variance.

### Canonical correlation analysis

**Figure 4:**
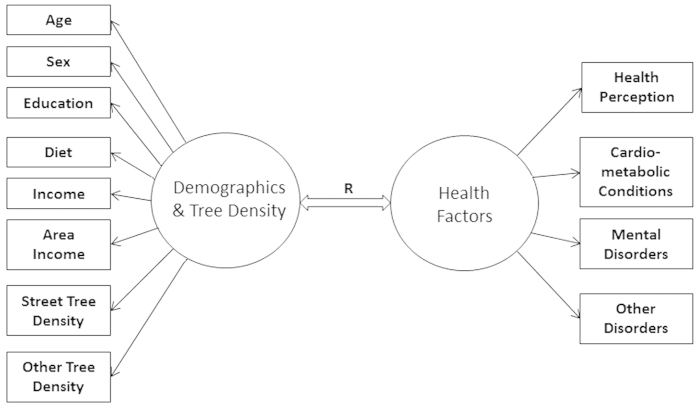
The canonical correspondence model that was used in our canonical correlation analyses to assess the relationship of the predictors (socio-economic, demographic and tree density variables) with health factors.

**Figure 5:**
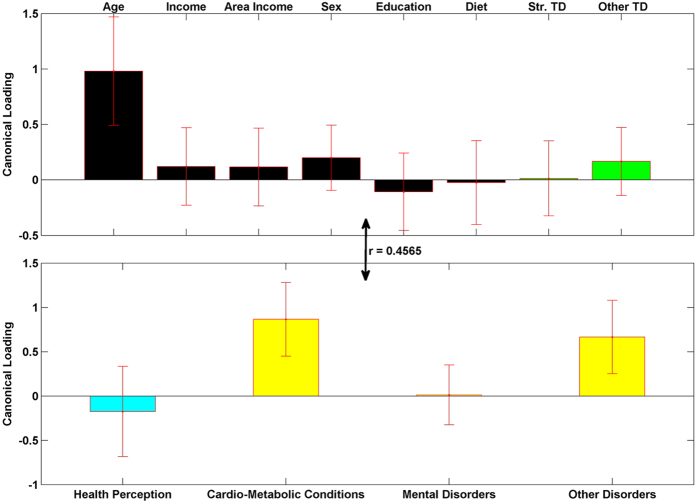
The first pair of linear composites for the canonical correlation analysis; F (32, 114680) = 381.2263), R^2^ = 0.2084, p < 0.0001. Bars show correlation of each variable (canonical loadings) with the first set of weighted canonical scores. Error bars show ±2 standard errors containing both between and within imputation variance calculated by bootstrapping imputed data sets. Please notice the different colors for health perception (teal) and other three health condition variables (yellow). This is to emphasize that they have different coding directions in terms of a person’s well-being (more health perception is positive, more health conditions is negative).

**Figure 6:**
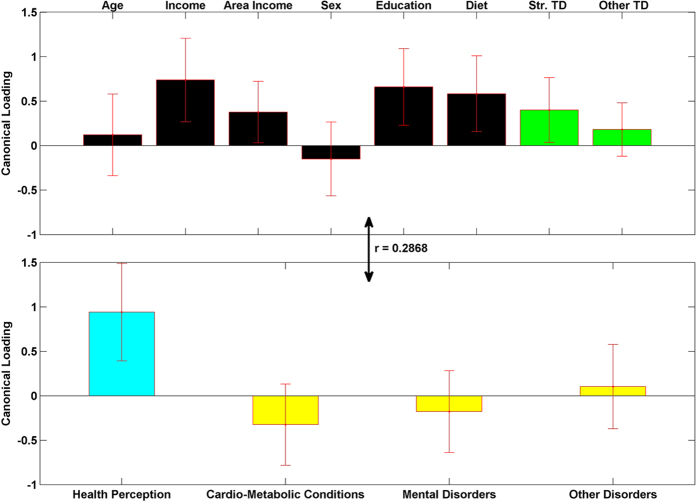
The second pair of linear composites for the canonical correlation analysis; F (21, 89297) = 211.0480), R^2^ = 0.0822, p < 0.0001. Bars show correlation of each variable with the second set of weighted canonical scores. Error bars show ±2 standard errors containing both between and within imputation. Please notice the different colors for health perception (teal) and other three health condition variables (yellow). This is to emphasize that they have different coding directions in terms of a person’s well-being (more health perception is positive, more health conditions is negative).

**Figure 7:**
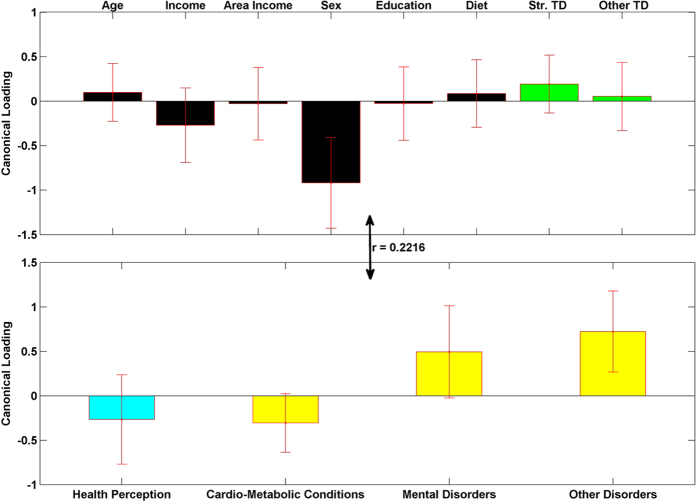
The third pair of linear composites for the canonical correlation analysis; F (12, 63702) = 139.9347, R^2^ = 0.0491, p < 0.0001. Bars show correlation of each variable with the third set of weighted canonical scores. Error bars show ±2 standard errors containing both between and within imputation variance. Please notice the different colors for health perception (teal) and other three health condition variables (yellow). This is to emphasize that they have different coding directions in terms of a person’s well-being (more health perception is positive, more health conditions is negative).

To investigate further the relationship between the two sets of variables, namely the health-related variables (Health Perception, Cardio-metabolic conditions, Mental Disorders, and Other Disorders) and the demographic and green-space variables (Age, Sex, Education, Income, Area income, Diet, Street Tree Density, and Other Tree Density), we performed a canonical correlation analysis^43^,^44^. Our model is presented in the diagram shown in Fig. 4. Mauchly’s test of sphericity was performed on the average of imputations in MATLAB (Sphertest: Sphericity tests menu) and showed that the correlation matrix of the data is significantly different from the identity matrix (p < 0.0001). This significant departure of the data from sphericity warrants the canonical correlation analysis.

In a canonical correlation analysis, first, the weights that maximize the correlation of the two weighted sums (linear composites) of each set of variables (called canonical roots) are calculated. Then the first root is extracted and the weights that produce the second largest correlation between sum scores is calculated, subject to the constraint that the next set of sum scores is orthogonal to the previous one. Each successive root will explain a *unique* additional proportion of variability in the two sets of variables. There can be as many canonical roots as the minimum number of variables in the two sets, which is four in this analysis. Therefore, we obtain four sets of canonical weights for each set of variables, and each of these four canonical roots have a canonical correlation coefficient which is the square root of the explained variability between the two weighted sums (canonical roots).

To obtain unbiased canonical weights for variables and canonical correlation coefficients, we averaged data values over the 30 imputations and performed canonical correlation analysis on the z-scores of the averaged data using MATLAB (MATLAB and Statistics Toolbox Release 2014a, The MathWorks, Inc., Natick, Massachusetts, United States). For a more straight-forward interpretation and better characterization of the underlying latent variable, instead of using the canonical weights, we calculated the Pearson correlation coefficient (canonical loading) of each observed variable in the set with the weighted sum scores for each of the four linear composites. This way, each canonical root (linear composite) could be interpreted as an underlying latent variable whose degree of relationship with each of the observed variables in the set (how much the observed variable contributes to the canonical variate) is represented by the loading of the observed variable and its errorbar (see canonical correlation results).

To estimate the standard errors of the canonical loadings, we bootstrapped z-scores from each of the 30 complete imputed data (1000 simulations for each) and performed canonical correlation analysis 30000 times using MATLAB. Then, we calculated the variances of the set of loadings, which were calculated as explained above, over each completed dataset (within imputation variance). We also calculated the variance of the 30 sets of coefficients (between imputation variance). The standard errors of the coefficients were then estimated using the same Rubin’s rules as was done for the regression analyses.

## Additional Information

**How to cite this article**: Kardan, O. *et al.* Neighborhood greenspace and health in a large urban center. *Sci. Rep.* **5**, 11610; doi: 10.1038/srep11610 (2015).

## Supplementary Material

Supplementary Information

## Figures and Tables

**Table 1:** **Combined results of regression of health perception on the multiply-imputed data**.

**Variable**	**Estimate**	**Std. Error**	**t-stat**	**p-value**	**df**	**Rel. Increase**	**FMI**
**Intercept**	2.7794	0.0296	93.8319	<0.0001	6202	0.0685	0.0644
**Diet**	0.0481	0.0024	19.7007	<0.0001	668	0.2130	0.1781
**Age**	–0.0059	0.0004	–16.8734	<0.0001	10566	0.05246	0.0500
**Sex**	0.0374	0.0107	3.4853	0.0004	14364	0.04498	0.0432
**Education**	0.0663	0.0032	20.6885	<0.0001	6647	0.06620	0.0624
**Income**	0.0710	0.0034	21.0145	<0.0001	448	0.2630	0.2117
**Area income**	0.0278	0.0056	4.9162	<0.0001	3664	0.08932	0.0825
**Street Tree den.**	0.0101	0.0015	6.6879	<0.0001	34158	0.02915	0.0284
**Other Tree den.**	–0.0003	0.0004	–0.7293	0.4658	25993	0.03342	0.0324

R^2^ = 0.0885, adjusted R^2^ = 0.0876, F (8, 7879*) = 94.6814, p < 0.0001. FMI is fraction of missing information. *The average of estimated degrees of freedom.

**Table 2:** **Combined results of regression of cardio-metabolic conditions on the multiple-imputed data**.

**Variable**	**Estimate**	**Std. Error**	**t-stat**	**p-value**	**df**	**Rel. Increase**	**FMI**
**Intercept**	0.1236	0.0363	3.4049	0.0008	895	0.1937	0.1643
**Diet**	–0.0062	0.0026	–2.3217	0.0204	1206	0.1569	0.1371
**Age**	0.0296	0.0004	70.4279	<0.0001	1724	0.1307	0.1166
**Sex**	0.2894	0.0128	22.5830	<0.0001	857	0.1871	0.1596
**Education**	–0.0570	0.0037	–15.2098	<0.0001	553	0.2351	0.1932
**Income**	–0.0240	0.0038	–6.2648	<0.0001	168	0.4563	0.3213
**Area income**	–0.0286	0.0066	–4.3071	<0.0001	863	0.1864	0.1591
**Street Tree den.**	–0.0097	0.0018	–5.4025	<0.0001	801	0.1937	0.1643
**Other Tree den.**	–0.0001	0.0005	–0.1196	0.9048	776	0.1970	0.1667

R^2^ = 0.1920, adjusted R^2^ = 0.1845, F (8, 871*) = 25.6089, p < 0.0001. FMI is fraction of missing information. *The average of estimated degrees of freedoms.

**Table 3:** **Communalities for the variables based on the canonical correlation analysis**.

**Variable**	**Communality**	**Variable**	**Communality**
Age	0.9845	Str. Tree Density	0.3980
Income	0.8158	Other Tree Density	0.1317
Area Income	0.2649	Health Perception	1.0000
Sex	0.9848	Cardio-metabolic Conditions	1.0000
Education	0.5016	Mental Disorders	1.0000
Diet	0.4372	Other Disorders	1.0000
