# Supplementary material for: Neighborhood greenspace and health in a large urban center
Source: Sci Rep. 2015 Jul 9;5:11610. doi: 10.1038/srep11610 (PMC4497305; doi:10.1038/srep11610)
Supplement: Supplementary Information [file srep11610-s1.doc]

**Supplementary Information for:**

**Neighborhood greenspace and health in a large urban center**

**Omid Kardan^1^, Peter Gozdyra^2^, Bratislav Misic^3^, Faisal Moola^4^, Lyle J. Palmer^5^, Tomáš Paus^6^ & Marc G. Berman^1^**

^1^Department of Psychology, The University of Chicago, Chicago, IL, USA

^2^Institute for Clinical Evaluative Sciences, Toronto, ON, Canada

^3^Indiana University, Bloomington, IN, USA

^4^The David Suzuki Foundation, Toronto, ON, Canada

^5^Translational Health Science, The University of Adelaide, Adelaide, SA, Australia

^6^Rotman Research Institute, University of Toronto, Toronto, ON, Canada

**Address correspondence to:**

**Omid Kardan or Marc G. Berman**

Department of Psychology

[University of Chicago](http://www.uchicago.edu/)

5848 S. University Avenue

Chicago, IL 60637

e-mail: [okardan@uchicago.edu](mailto:okardan@uchicago.edu)

or

[bermanm@uchicago.edu](mailto:bermanm@uchicago.edu)

**Supplementary Tables**

| **Health Condition** | **% Yes Response** | **Health Condition** | **% Yes Response** |
| --- | --- | --- | --- |
| Anxiety | 5.6% | Cirrhosis | 0.3% |
| Addiction | 1.1% | Colitis | 0.9% |
| Depression | 9.8% | COPD | 0.9% |
| Blood Glucose | 4.2% | IBD | 6.0% |
| Diabetes | 4.5% | Cancer | 7.5% |
| Hypertension | 16.4% | Migraines | 9.2% |
| High Cholesterol | 11.9% | Sleep Apnea | 5.6% |
| MI (heart attack) | 1.4% | Arthritis | 14.8% |
| Stroke | 0.8% | Asthma | 13.1% |
| Heart Disease | 2.7% | None | 33.7% |

Table S1. Percentage of respondents reporting health conditions (%Yes responses)

| **Variable** | **Fraction Missing** | **Variable** | **Fraction Missing** |
| --- | --- | --- | --- |
| Age | 0.000 | Diabetes | 0.032 |
| Sex | 0.000 | Obesity | 0.167 |
| Alcohol Frequency* | 0.115 | MI | 0.029 |
| Smoking* | 0.023 | Heart Disease | 0.207 |
| Education | 0.031 | Cancer | 0.024 |
| Income | 0.158 | Migraines | 0.350 |
| Area Income | 0.034 | Asthma* | 0.035 |
| Walking* | 0.105 | Arthritis | 0.038 |
| Fruit Number | 0.138 | Cirrhosis* | 0.026 |
| Vegetable Number | 0.104 | IBD* | 0.036 |
| Alc. Binge* | 0.166 | Colitis* | 0.028 |
| Population density* | 0.011 | COPD* | 0.039 |
| Health perception | 0.013 | Anxiety | 0.364 |
| Blood Glucose | 0.355 | Addiction | 0.353 |
| High BP | 0.038 | Depression | 0.048 |
| High Cholesterol | 0.351 | Other Tree Density | 0.000 |
| Stroke | 0.031 | Str. Tree Density | 0.000 |

Table S2. The fractions of missing data for the variables used in the multiple imputations. *These variables were only used in the imputation analysis and not in the analyses.

| Variable | Estimate | Std. Error | t-stat | p-value | df | Rel. Increase | FMI |
| --- | --- | --- | --- | --- | --- | --- | --- |
| **Intercept** | 0.4187 | 0.0208 | 20.8755 | <0.0001 | 254 | 0.3592 | 0.2700 |
| **Diet** | -0.0018 | 0.0016 | -1.1215 | 0.2627 | 104 | 0.6216 | 0.3948 |
| **Age** | 0.0011 | 0.0002 | 4.6133 | <0.0001 | 197 | 0.4116 | 0.3009 |
| **Sex** | -0.0837 | 0.0078 | -10.7144 | <0.0001 | 415 | 0.2741 | 0.2189 |
| **Education** | -0.0028 | 0.0022 | -1.2688 | 0.2049 | 213 | 0.3969 | 0.2907 |
| **Income** | -0.0241 | 0.0022 | -10.9918 | <0.0001 | 286 | 0.3358 | 0.2566 |
| **Area income** | -0.0140 | 0.0039 | -3.5579 | 0.0004 | 210 | 0.4003 | 0.2925 |
| **Street Tree den.** | 0.0068 | 0.0011 | 6.0495 | <0.0001 | 613 | 0.2228 | 0.1849 |
| **Other Tree den.** | -0.0004 | 0.0003 | -1.5259 | 0.1543 | 327 | 0.3120 | 0.2424 |

Table S3. Combined results of regression of mental disorders on the multiply-imputed data. R^2^ = 0.0136, adjusted R^2^ = -0.0111, F (8, 319*) = 0.5503, p=0.1820. FMI is fraction of missing information. * The average of estimated degrees of freedoms.

| Variable | Estimate | Std. Error | t-stat | p-value | df | Rel. Increase | FMI |
| --- | --- | --- | --- | --- | --- | --- | --- |
| **Intercept** | 0.1863 | 0.0256 | 7.2689 | <0.0001 | 276 | 0.3427 | 0.2605 |
| **Diet** | 0.0047 | 0.0021 | 2.2509 | 0.0252 | 235 | 0.3749 | 0.2787 |
| **Age** | 0.0127 | 0.0003 | 42.4625 | <0.0001 | 343 | 0.3040 | 0.2376 |
| **Sex** | -0.2194 | 0.0088 | -24.8472 | <0.0001 | 723 | 0.2044 | 0.1720 |
| **Edu** | -0.0107 | 0.0026 | -4.1323 | 0.0002 | 348 | 0.3015 | 0.2360 |
| **Income** | -0.0124 | 0.0028 | -4.4833 | <0.0001 | 322 | 0.3146 | 0.2440 |
| **Area income** | -0.0117 | 0.0048 | -2.4364 | 0.0229 | 827 | 0.1905 | 0.1621 |
| **Street Tree den.** | 0.0064 | 0.0013 | 4.9644 | <0.0001 | 159 | 0.4728 | 0.3294 |
| **Other Tree den.** | 0.0005 | 0.0003 | 1.4618 | 0.0988 | 250 | 0.3618 | 0.2715 |

Table S4. Regression results of Other Disorders: R^2^ = 0.1167, adjusted R^2^ = 0.0980, F (8, 387*) = 6.2397, p<0.0001. * The average of estimated degrees of freedoms. FMI is fraction of missing information.

**Supplementary Figures**


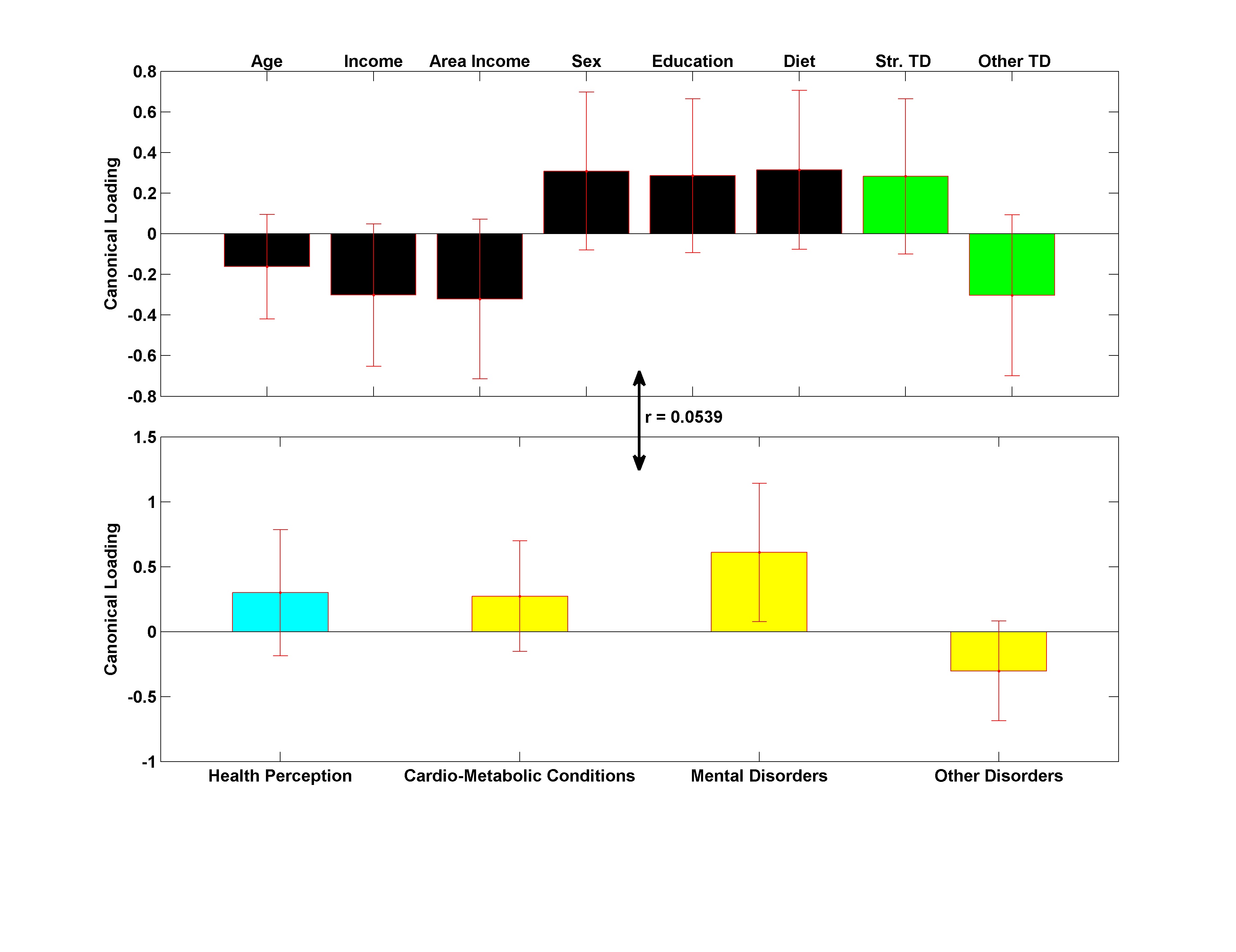


Figure S1. The fourth pair of linear composites for the canonical correlation analysis; F (5, 31852) = 18.0958), R^2^ = 0.0029, p<0.0001. Bars show correlation of each variable with the fourth set of weighted canonical scores. Error bars show ±2 standard errors containing both between and within imputation.

**
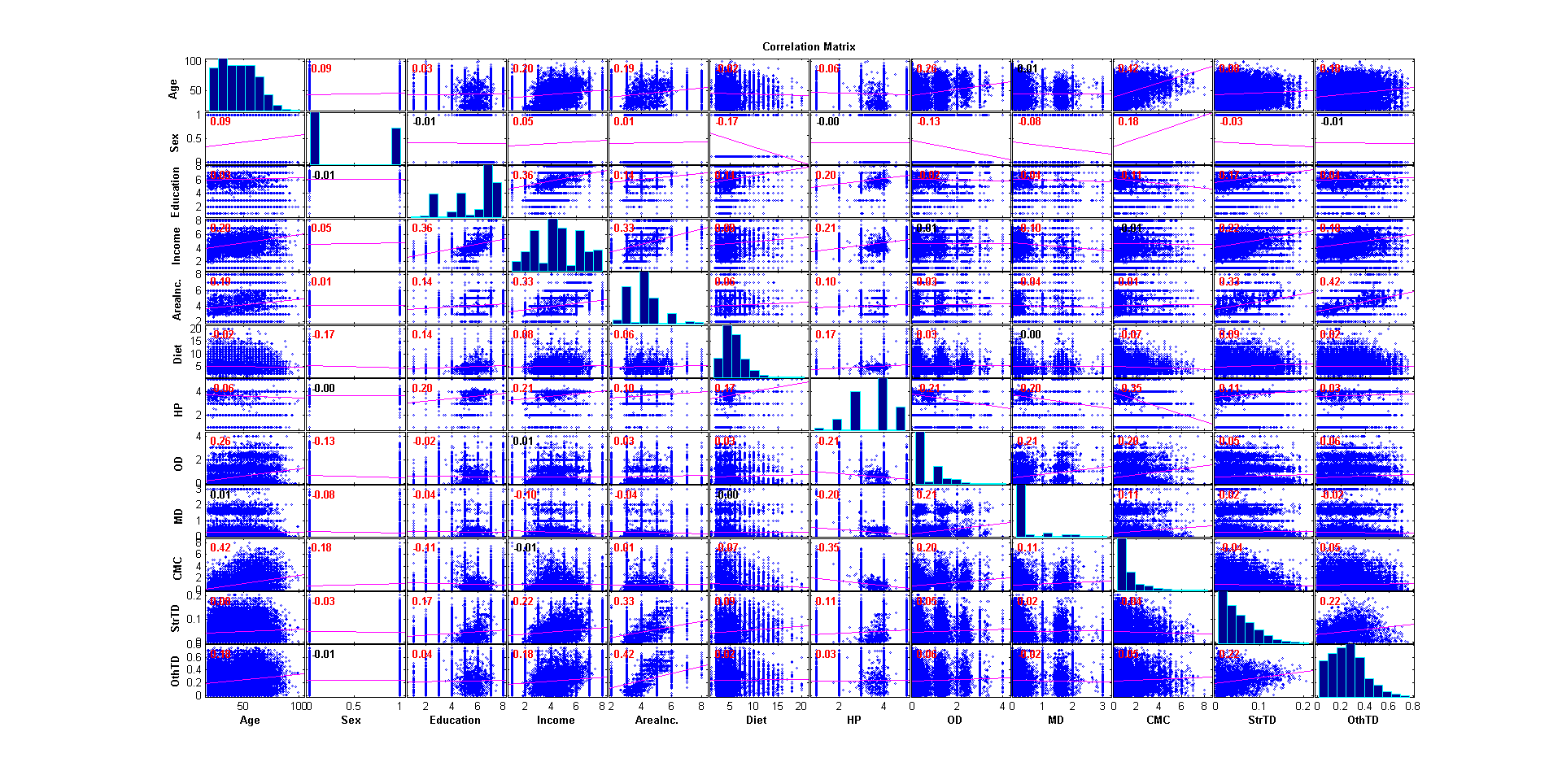
**

Figure S2. Correlation matrix of demographic variables, health outcomes, and tree density variables. Each scatterplot square shows the correlation between variables that correspond to the row and column of the square and the significance (p-value) of that correlation is indicated in the color of the correlation coefficient: red means p <0.05, and black means p> 0.05. The histograms on the main diagonal of the matrix show the distribution of the data for the corresponding variable. HP is Health Perception, OD is Other Disorders, MD is Mental Disorders, CMC is Cardio-Metabolic Conditions, strTD is street Tree Density, othTD is other Tree Density.

**Supplementary Equations**

Relationships between crown diameter and DBH:

Length measures (dbh and crown diameter) are in meters. Numbers in [ ] show the reference from which the formula was extracted (some of the coefficients are changed due to converting the original formula to metric measures).

Crown diameter (Maple) = 0.3048*(-0.543+4.691*(dbh*0.3937)^0.688^) Ref. [36]

Crown diameter (Locust) = 0.007+0.825*log (dbh)+0.077*log(dbh)^2^ Ref. [36]

Crown diameter (Spruce/Pine) = 0.3048*(1.634+3.628*(dbh*0.3937)^0.723^) Ref. [36]

Crown diameter (Ash) = 0.3048*(-7+7.72*(dbh*0.3937)^0.589^) Ref. [36]

Crown diameter (Linden) = 0.3048*(-1.4+4.302*(dbh*0.3937)^0.667^) Ref. [36]

Crown diameter (Cherry) = 1.76+0.1540*dbh Ref. [35]

Crown diameter (Oak) = 1.717+0.156159*dbh Ref. [35]

Crown diameter (Birch) = 0.975+0.161512*dbh Ref. [35]
